# Exemplary Care among Chinese Dementia Familial Caregivers

**DOI:** 10.3390/healthcare6040141

**Published:** 2018-12-05

**Authors:** Bobo Hi Po Lau, Vivian Weiqun Lou, Karen Siu Lan Cheung

**Affiliations:** 1 Department of Counselling and Psychology, Hong Kong Shue Yan University, Hong Kong, China; 2 Sau Po Centre on Ageing, University of Hong Kong, Hong Kong, China; 3 Department of Social Work and Social Administration, University of Hong Kong, Hong Kong, China; wlou@hku.hk; 4 Mindlink Research Centre, Hong Kong, China

**Keywords:** quality of care, family, informal care, dementia, Alzheimer’s disease, Chinese

## Abstract

Objective: This study investigates the feasibility of using the Exemplary Care Scale (ECS) among Chinese dementia familial caregivers, and reports its psychometric properties. Method: Back translation was used to develop the Chinese version of ECS (C-ECS). Three hundred and ninety-seven dyads of caregivers and their relatives with dementia responded to an assessment battery which included questions on care recipients’ cognition, behavioral and psychological symptoms, daily activities assistance, social support, and caregiver well-being. Results: Results of an exploratory principal component analysis revealed two subscales in the 11-item C-ECS: considerate caregiving and preserving esteem. C-ECS and its subscales demonstrated sufficient reliability, as well as criteria-related validity through its association with care recipient’s cognition and health, and caregivers’ well-being and social support. Discussion: Our findings provide preliminary support to C-ECS as a reliable and valid measure of exemplary caregiving among Hong Kong Chinese familial dementia caregivers. In the light of the increasing importance of familial care in dementia care planning, we recommend the use of this brief scale in regular caregiver assessment in research and service delivery.

## 1. Introduction

Familial care has long been the backbone of long-term care for older adults with physical and cognitive impairment [1]. Much care work, rendering assistance to the frail and dependent members of families, is informal, performed at home by untrained, unpaid family members who are often daughters or wives [2]. Compared to formal care providers, familial caregivers are assumed to be more familiar with their relatives with dementia, and tend to provide care that better fits the preferences and values of their relatives. Despite this widely-held assumption, familial care may not always meet the physical and psychosocial needs of the person with dementia (PWD). Furthermore, a low quality of home care and adverse events such as falls and infections, have been found to increase the risk of long-term care placement and, indirectly, shorten survival [3] and recurrent hospitalization [4]. Hence, it is paramount to assess the quality of familial care for profiling cases, matching services, promoting good practices and program evaluation.

Assessments for person-centered, high-quality care for PWD tend to concentrate on institutional contexts (e.g., nursing home, hospitals) and clinical settings [5]. Yet, the assessment of home care is largely lacking [6]. The quality of familial care for elderly has been traditionally measured by the absence of aggression such as abuse [7,8,9] and potentially harmful behaviors (PHB) [10,11] toward the care recipients, or the fulfillment of needs for assistance on activities of daily living (ADL) [12,13]. Providing assistance for ADL can result in a burden and agitation or uplifts and positive outcomes [14]. However, adequate ADL assistance and the absence of aggressive behaviors tend to indicate care of only a very basic standard. Such basic care is unlikely to sufficiently reflect the meaning of quality familial care according to the society, the caregivers, and the care recipients. On the other hand, familial care that is perceived to be overly protective, insensitive, or inappropriate by the care recipients tends to backfire, and is prospectively associated with more depressive symptoms in both caregivers and recipients [15]. 

With respect to the difference between basic and quality familial care, Dooley and colleagues [16] reintroduced the concept of exemplary care (EC). It refers to care that shows caregivers’ willingness and passion in providing care beyond the bounds of fulfilling the basic needs of the elderly, demonstrates respect for the feelings, preferences, opinions, and values of the elderly, and avoids denigrating or criticizing their limitations. According to the person-centered framework which conceptualizes dementia caregiving as involving a meaningful and dignified relationship between the caregiver and the PWD [17,18], the quality of the dyadic relationship, whether it is warm or conflictual, should constitute an important component for the assessment of care quality [19]. When developing a good quality management plan for PWDs, understanding the experiences and perspectives of PWDs and their caregivers is important in providing optimal care [20]. Although there are instruments to measure knowledge and skillsets of family caregivers of PWDs [21,22], these instruments could be inadequate in capturing the person-centeredness of the caregiver–care recipient relationship that characterizes EC in dementia caregiving.

To measure exemplary care, the 11-item Exemplary Care Scale (ECS) has been developed from the Family Relationships in Late Life Project, in which 41% of care recipients were suffering from dementia [16]. Factor analyses indicated that the ECS consists of two factors reflecting the provision of exceptional and personalized care (Provide) and the respect for care-recipient’s feelings, opinions, and values (Respect). Nonetheless, Harris and colleagues [23] found only a single factor using the Reaching out Dementia Caregiver Support Project (REACH-II) dataset with all PWD cases. Despite the disagreement on factor structure, a higher level of EC has been repetitively found to be associated with less suicidal ideation and greater perceived control over health outcomes among PWDs, less depression, resentment, burden, bother with behavioral and psychological symptoms of dementia (BPSDs), desire of institutionalization and more positive aspects of caregiving (PAC) among caregivers, and fewer PHBs in the caregiving relationship [16,23,24,25]. In brief, EC tends to be an antidote to harmful caregiving behaviors and beneficial to the well-being of both PWDs and caregivers. 

In response to the increasing demand to capture a high quality of familial care in cross-cultural research and local practice [1], this article reports the first attempt to investigate the factor structure and criteria-related validity of the Chinese version of ECS (C-ECS) among Hong Kong Chinese familial dementia caregivers. In light of previous findings with ECS [16,23,24,25], we expected C-ECS and its subscales to show criteria-related validity by relating negatively with the occurrence of BPSDs, and positively with cognition and caregiver-rated health among PWDs. We also expect C-ECS to be associated with higher levels of PAC, confidence in dealing with BPSDs, and social support, and lower levels of depression, caregiving burden, bother with BPSDs, assistance and frustration with ADLs, Instrumental ADLs (IADLs), and PHBs.

## 2. Methods

### 2.1. Recruitment and Procedures

Participants were caregivers who participated in a non-pharmacological, multidimensional caregiver intervention program adopted for Hong Kong Chinese (Reaching out Dementia Caregiver Support Project; REACH-HK) [26]. We used the data collected at the baseline assessment for the current analysis. The inclusion criteria for caregivers included: (a) being 21 years old or above; (b) providing care or accompaniment to a family member medically diagnosed with dementia for no less than two hours per day for at least six months before recruitment; (c) with no plans of leaving Hong Kong during the period of intervention and assessment; and (d) self-reporting the presence of at least two of the following symptoms of caregiver stress in the past month: feeling exhausted, tearful, angry and frustrated, stressful, and disconnected from friends or relatives, and experiencing deteriorating health. Caregivers was excluded if they were either: (a) non-Chinese or non-Cantonese speaking; (b) participating in an active cancer treatment; (c) with impending plans to institutionalize PWD in six months; (d) participating in another clinical trial or structured non-pharmacological intervention; or (e) were cognitively impaired (Short Portable Mental Status Questionnaire score ≤ 4). Participants were recruited from 61 elderly service units of 11 local non-governmental organizations (NGOs). The data were collected through face-to-face interviews with caregivers conducted by trained registered social workers at caregivers’ homes or counseling rooms of their respective elderly service centers. Written informed consent was obtained from participants prior to any assessments or intervention. 

### 2.2. Developing the Chinese Version of Exemplary Care Scale (C-ECS)

#### 2.2.1. Translation and Back Translation

The development of the 11 English items has been documented in Dooley et al. [16]. A bilingual research assistant first translated the English items into Cantonese, the predominant Chinese dialect used by caregivers in Hong Kong and of Guangdong origin. Another bilingual research assistant then translated the Cantonese items into another set of English items (i.e., back translate [27]). The two research assistants were trained in nursing and psychology, respectively. The principal investigator evaluated the quality of the translation by assessing the resemblance of the original set and the back-translated set of English items, as well as the grammatical accuracy and eloquence of the Cantonese items. 

#### 2.2.2. Expert Validation on Face Validity

The frontline social workers of the program, the steering committee, and the multidisciplinary research team with experts in demography, clinical psychology, gerontology, social work, and nursing inspected the Cantonese items. The inspection began by circulating the questionnaire through emails followed by a face-to-face meeting. They evaluated the items’ relevance to an exemplary quality of familial care within the caregiving context in Hong Kong, and whether the items were phrased in a manner easily understood by middle-aged and elderly caregivers. Face validity of the items was established by the reassurance from the frontline social workers who were going to be involved in the trial and be the assessors who would use the items. The expert team agreed that the items were easy to comprehend and administer. 

#### 2.2.3. Piloting

We piloted the translated items on 24 caregivers of similar demographic and caregiving backgrounds as the current sample during the pilot trial of REACH-HK. They reported neither difficulties with administering the translated scales, nor rejections from participants toward the items. 

Participants were instructed to evaluate the frequency which they have practiced the 11 items during their recent care for PWDs, and responded on a 4-point scale (0 = *never*, 1 = *sometimes*, 2 = *often*, 3 = *always*). 

### 2.3. Measures to Test Criteria-Related Validity

#### 2.3.1. Care Recipient Characteristics

BPSDs were assessed by three single-item questions (memory-related, disruption-related, and mood-related symptoms) adapted from the assessment package of an abridged, translated REACH-II like intervention [28] and the Revised Memory and Problem Behavior Checklist used in REACH-II [29]. The frequency of occurrence of these three categories of symptoms in the previous week was evaluated. Cognition was evaluated by the Cantonese version of the Mini Mental State Examination (MMSE) [30]. Caregivers were also asked to rate the physical health of PWDs by a single question on a five-point scale in spite of their memory impairment. 

#### 2.3.2. Caregiver Characteristics

The 20-item Center for Epidemiologic Studies Depression Scale (CESD) [31] (Cronbach α = 0.85) and the 12-item Zarit Burden Interview (ZBI) [32] (Cronbach α = 0.84) were adopted to measure depressive symptoms and caregiving burden respectively. The 11-item Positive Aspects of Caregiving scale (PAC) [33] (Cronbach α = 0.89) was used to measure positive caregiving role appraisals. Bother and confidence in dealing with each category of BPSDs (memory-related, disruption-related, and mood-related symptoms) were assessed by three single-item questions. The number of instrumental ADLs (telephoning, grocery shopping, preparing meals, simple housework, washing clothes, using public transport, medication, managing finances; [34], Cronbach α = 0.78) and ADLs (indoor transfer, eating, bathing, wearing clothes, toileting, grooming; [35], Cronbach α = 0.85) the caregivers had assisted in the previous week were tallied. Caregivers’ levels of frustration for each IADL and ADL (for the task where the caregiver has not involved, the frustration score was coded as zero) were averaged to yield the frustration scores for the two types of assistance (IADL frustration: Cronbach α = 0.84; ADL frustration: Cronbach α = 0.82). Social support was measured by four items covering informational support, accompaniment, emotional support, and isolation (Cronbach α = 0.63). Caregivers’ PHB were recorded by two items, including “How often in the past six months, have you felt like screaming or yelling at (PWD) because of the way he/she behaved”, and “How often in the past six months, have you had to keep yourself from hitting or slapping (PWD) because of the way he/she behaved”. 

Demographic characteristics of caregivers and PWDs (e.g., age, gender, caregiving years irrespective of diagnosis, etc.) were also obtained. 

### 2.4. Data Analyses

We conducted an exploratory principal component analysis with promax rotation [36] on the 11 C-ECS items. The extraction of components was guided by multiple criteria [36,37], including the results of the scree plot [38], eigenvalues (minimum eigenvalue for extraction = 1) [39], and the meaningfulness of components. Reliability of the scale and subscales was assessed by Cronbach’s alphas. Pearson’s correlations were used to analyze relationships between continuous variables (e.g., C-ECS); while t-tests were used to assess associations between a continuous variable and a binary categorical variable (e.g., gender). Statistical analyses were conducted with SPSS version 19.0.0. (Armonk, NY, USA).

### 2.5. Ethical Approval

This study was approved by the Human Research Ethics Committee for Non-Clinical Faculties of the University of Hong Kong (Reference Number: EA270811).

## 3. Results

Four hundred and fifty-six caregivers were eligible for the intervention. Only data from participants with valid answers on all 11 EC items were included in the current analysis, resulting in a sample size of 397 (87.1%). (Forty-two (9.2%) participants missed one item on the 11-item ECS, eight (1.8%) participants missed two items. Another eight participants (1.8%) missed three items. Only one participant missed four items. 397 (87.1%) participants provided valid answers for all 11 ECS items.) Table 1 presents the sample characteristics. Compared to the participants included in the analysis, the excluded group was less likely to have attained more than primary education, χ^2^(1) = 5.00, *p* = 0.025. Other demographic attributes were comparable across the two groups.

### 3.1. Item Responses

Table 2 presents the descriptive statistics of the items. The most endorsed items were BRIGHT ENVIRONMENT, GROOM, and RELAXED ATMOSPHERE. The mean scores were 2.34, 2.19, and 2.14 respectively, which fell between the response categories of “*often*” and “*always*”. The least endorsed items were NOT TREATING CHILD-LIKE, SIT AND TALK, and NOT OVERCRITICAL. The mean scores were 1.26, 1.51, and 1.68 respectively, which fell between “*sometimes*” and “*often*”. The items were not significantly skewed (mean skewness = −0.33).

### 3.2. Component Structure

The current sample has fulfilled the prerequisites for conducting principal component analysis, as shown by the Kaiser-Meyer-Olkin Measure of sampling adequacy (0.88) and Bartlett’s test of sphericity results, χ^2^(55) = 1043.03, *p* = 0.000. Agreeing with the point of inflexion on the scree plot, two components of eigenvalues over 1 (4.04 and 1.04) were extracted, explaining 46.1% of the total variance. The first extracted component encompasses five items. The minimum component loading was 0.53 (HOBBY). The second extracted component comprises the rest of the six items, with a minimum component loading of 0.37 (GATHERING). Table 2 also tabulates the component loadings.

The two extracted components respectively resembled *provide* and *respect* on Dooley et al. (2007). However, GATHERING, which originally belonged to *provide*, was found to load relatively better on the second component. In addition, HOBBY and RELAXED ATMOSPHERE tended to load favorably on the first component, instead of the second component as predicted by Dooley et al. [16], HOBBY and RELAXED ATMOSPHERE tend to imply care that makes deliberate and considerate arrangements to accommodate the psychological needs of PWDs for their engagement and peace of mind. Therefore, instead of adopting the term *provide* for the first component, we named the component “*Considerate caregiving*”. GATHERING was loaded on the second component together with the remaining items on respect of Dooley et al. Because HOBBY and RELAXED ATMOSPHERE have been moved to “*Considerate caregiving*”, we reckon that the second component tends to describe care that conveys respect for the valuable role of the PWD in the family. Therefore, we labelled the second component “*Preserving esteem*”.

The Cronbach’s alpha of C-ECS was 0.81, suggesting satisfactory reliability in the current sample. The corresponding estimates for *considerate caregiving* and *preserving esteem* were 0.78 and 0.66, respectively, which indicate sufficient reliability. The two subscales were strongly correlated to each other (*r* = 0.60). We derived the scale score by summing up responses on the 11 items. The resultant range runs from 0 to 33. C-ECS has a mean of 20.50, with a standard deviation of 6.07. The scale was not significantly skewed (skewness = −0.029, *SE* = 0.12). Considerate caregiving has a score range of 0 to 15, and a mean (standard deviation) of 10.86 (3.15), with five items. The score range of Preserving esteem is 0 to 18, with six items. The mean (SD) was 9.64 (3.65).

### 3.3. Criteria-Related Validity

Table 3 shows the inter-correlations of C-ECS and its subscales with the occurrence of BPSDs, cognition, and caregiver-rated health of PWDs. C-ECS and its subscales were not significantly associated with the occurrence of BPSDs. However, higher scores of C-ECS and its subscales were related to better caregiver-rated health. *Preserving esteem* was also positively associated with MMSE scores.

Table 4 displays the inter-correlations of EC and its subscales with a collection of caregiver characteristics. All three scales of EC were related to less depressive symptoms and bother with memory-related symptoms, and higher levels of PAC, confidence in dealing with memory-related and mood-related symptoms, and social support. Only C-ECS and *preserving esteem* were related to lower levels of caregiving burden, bother with disruption-related symptoms, and lower levels of both types of PHBs. Also, only *preserving esteem* was found to be associated with lower IADL load, and frustration with IADL and ADL assistance.

C-ECS and its subscales were related to a shorter period of caregiving history (C-ECS: *r* = −0.14, *p* = 0.015; *Considerate caregiving*: *r* = −0.12, *p* = 0.039; *Preserving esteem*: *r* = −0.13, *p* = 0.029). C-ECS and *considerate caregiving* scores were higher among dyads with PWDs having secondary school education or above (C-ECS: *M* = 21.68, *SD* = 5.99; *Considerate caregiving*: *M* = 11.53, *SD* = 3.01) than among cases with PWDs with no schooling or only primary education (C-ECS: *M* = 20.15, *SD* = 6.14, *t*(382) = 2.51, *p* = 0.013; *Considerate caregiving*: *M* = 10.64, *SD* = 3.21, *t*(382) = 2.21, *p* = 0.028). Other demographic variables were not significantly related to the levels of C-ECS and its subscales.

## 4. Discussion

This study investigates the feasibility of using ECS among Chinese dementia familial caregivers, and reports on its psychometric properties. Our findings provide preliminary support to C-ECS as a reliable and valid measure of exemplary care for profiling cases, matching services, and program evaluation in research and practice settings with Chinese caregivers.

### 4.1. Reponses on Items and Factor Structure

The factor loadings of C-ECS were different from those of Dooley et al. [16] and Harris et al. [23]. We contend that compared to *Provide* of Dooley et al. [16], *Considerate caregiving* which included RELAXED ENVIRONMENT and HOBBY tends to emphasize a caregiver’s considerate and intentional attempt to create a psychologically calming and pleasantly caring environment for PWDs, in addition to providing adequate daily living assistance. *Preserving esteem*, which encompasses GATHERING, tends to indicate respect to the seniority and value of the cognitively-impaired elderly in family and social circles. These differences in factor loadings may reflect meaningful cultural differences in the meaning of exemplary care behaviors from caregivers’ perspective. For instance, involving the PWDs in familial gatherings despite their cognitive impairment could be an act of paying reverence to their seniority as well as cherishing their presence in the family [40], more so than a means to provide sufficient care among Chinese families. HOBBY and RELAXED ATMOSPHERE were perceived as a part of an exemplary caregiving routine, rather than a means to show respect among our sample. The Chinese culture expects the younger generation to provide all-rounded, instrumental support to their elderly, such as financial provision, scheduling activities, transportation, handling household chores, etc. [41]. The independence of the elderly is relatively down-played under such cultural milieu, compared to the situation in an individualistic culture. Thus, maintaining the elder’s hobbies and providing an equanimous environment fit into the daily care routine of an exemplary Chinese caregiving condition.

In summary, the two factors—Considerate caregiving and Preserving esteem—reflect culturally important dimensions of elderly care. Chow [41] postulates three levels of filial practices of the Chinese culture, with the first level emphasizing satisfaction of physical needs and comforts, the second level stipulating the attention paid to parents’ preferences and wishes, and the third level indicating fulfilling the needs for respect and esteem of the parents. While the former factor, which captures the all-roundedness of exemplary practical care, fulfills the first two levels of filial practices, the latter factor highlights the importance of revering the seniority of the PWD in the hierarchical family system and fulfill the third level.

### 4.2. Criteria-Related Validity

The current findings also illuminate the association between cognitive capacity of PWD and the quality of care. *Preserving esteem* was related to a higher level of cognitive functioning and more favorable caregiver-rated health. Caregivers tended to exhibit greater *considerate caregiving* to better educated PWDs, too. Kitwood [17] has remarked the difficulties for caregivers to treat PWDs consistently as dignified persons, and the caregiving relationship as mutually meaningful. As reflected from the higher endorsement on items of *considerate caregiving* relative to those of *preserving esteem*, there may be a gradation of difficulties in practicing various types of EC, possibly with behaviors that preserves a high level of personhood and self-dignity being more difficult than those requiring greater attention to daily caregiving routines. Treating a cognitively impaired elderly person in a child-like manner can be considered as a form of elder abuse [42], and tends to be as common in the Chinese context as in Western societies [43]. Infantilization could be used to offset emotional distress in caregiving or rendering forgiveness to unpleasant and uncontrollable behaviors of PWDs easier. Further investigation is needed to explore how and why caregivers infantilize PWDs in the Chinese context.

Agreeing with previous findings on the association between the use of encouragement and supporting strategies (e.g., praising PWD, getting PWD to discuss feelings) and caregiver role adjustment [44,45], EC was related to more PAC, confidence, and social support, and less depression, burden, and PHBs among our Chinese caregivers. Surprisingly, EC was unrelated to the amount of BPSDs exhibited, but to the degree of bother with BPSDs and frustration with IADL and ADL assistance. This may imply that changing attitudes to the disease and the caregiving role may facilitate EC, even when BPSDs are relatively common and the caregiving load is high. *Considerate caregiving* was not as robustly related to the appraisals of BPSDs and caregiving tasks as *preserving esteem*. In this light, we suggest caregiver interventions to teach beyond “hard” caregiving skills (e.g., bathing, feeding), but also enable caregivers to adopt a “soft” and more person-centered view toward PWDs [19]. Caregivers may also refer to the items on C-ECS for a culturally-valid reference on quality home care.

Although C-ECS scores were statistically related to a multitude of caregiving outcomes and indicators of PWD health, the strength of associations remains modest [46]. This highlight the extensiveness of repertoire of factors affecting caregiver role adjustment, including contextual factors, personal and interpersonal resources [47]. This also suggest that EC could be independent of the condition of the PWD, and that EC could be an achievable quality regardless of the severity of the disease.

### 4.3. Limitations and Future Directions

The current study possesses several limitations. First, this study lacks PWD-rated or professionals-rated quality of care data to cross-validate the current caregiver-rated care quality. Future studies may characterize the care quality of a case from perspectives of multiple stakeholders, for instance, by applying C-ECS alongside nurse-rated scales such as QUALCARE [48] or by incorporating specific patient- and caregiver-centered goals for care into residential and clinical settings as the disease progressed [49]. We also encourage future studies to adopt qualitative and quantitative methods to explore the lay theories of quality familial care in different cultures, and compare their findings with the current results.

In addition, C-ECS provides a more comprehensive assessment of the quality-of-care continuum, and is useful to familial caregivers and volunteers, but also to researchers and healthcare professionals who are interested in quality of informal care and should be considered linking to a Social Ecological Model (the C3P Model) [50], the Information Communication Technology-based framework such as eCare solutions in the DEDICATE architecture service delivery [51] and dementia palliative and end-of-life care approach [52,53,54] and to continual improvement in care and education in clinical and care settings such as the Exemplary Care and Learning Site (ECLS) model [5] so that better patient outcomes, notably quality-of-life, and the full spectrum of person-centered services with lower costs, can be delivered to these patients and their families across an individual’s whole dementia journey.

Third, because this study relied on cross-sectional data, the direction of causality can hardly be inferred. On the one hand, EC could be the outcome of favorable personal and social resources [55], such a high level of positive role appraisals and social support, and low emotional distress. On the other hand, the practice of EC could be conducive to a loving and harmonious dyadic relationship, which may foster the well-being of caregivers and PWDs [15,24].

Fourth, although the research assistant was proficient in both English and Cantonese, we did not employ a professional translation service for checking the linguistic accuracy of the items. Future validation studies are encouraged to utilize professional translation services in this regard.

Fifth, we did not explicitly engage the caregivers in reviewing each of the items. Instead, our caregivers were asked to review the intervention trial at-large and the instrument as one of the components only. Future validation studies may consider explicitly asking caregivers for their ratings and comments to strengthen the validity of the translated or constructed items.

Moreover, as we utilized the baseline dataset of a single-arm intervention trial for investigating the psychometric properties of C-ECS, we could not study the test-retest reliability of the scale. The use of the post-intervention follow-up data would not be appropriate. Since the participants would have gone through a structured intervention, the variance of their levels of exemplary care would likely be reduced. The use of a multiple baseline design would also introduce unnecessary assessment burden and lengthen the waiting time for service among our strained caregivers who were looking forward to a structured psychosocial intervention. We regard the lack of testing of the test-retest reliability a limitation of this study and encourage future studies to investigate the temporal stability of EC using longitudinal methods and less stressful participants.

Lastly, although the C-ECS items and scales were not significantly skewed and the answers on all C-ECS items covered a full range of response options, participants were awaiting an intensive individualized intervention program conducted by the assessor while completing the baseline assessment. The concern for social desirability may have potentially resulted in biased judgment of the frequency of EC behaviors.

## 5. Conclusions

In the light of the important role of familial care in dementia care planning, the need for assessing an exemplary quality of familial care in research and practice is paramount. This study reported the psychometric properties of the C-ECS among Hong Kong Chinese familial dementia caregivers. Our results illustrate the psychometric properties of C-ECS and provide preliminary support to the instrument as a potentially reliable and valid measure for an exemplary quality of familial care.

## Figures and Tables

**Table 1:** Characteristics of sample (N = 397).

Characteristics	Value
CG Age in years, *M*/(*SD*)/Range	63.3/(12.7)/23–90
CG Female, n (%)	307 (77.3)
CG Married, n (%)	288 (72.5)
CG Educational Attainment, n (%)	
No schooling	38 (9.8)
Primary	119 (30.7)
Junior secondary	80 (20.6)
Senior secondary or above	151 (38.9)
CG’s Relationship with CR, n (%)	
Spouse	198 (50.2)
Child/child-in-law	192 (48.5)
Others	5 (1.3)
CG Job Status, n (%)	
Home-makers/Retired	267 (67.3)
Full-time/Part-time job	100 (25.2)
No work, not retired	30 (7.5)
Years of caregiving, *M*/(*SD*)/Range	5.8/(7.4)/0.5–60
CR Age in years, *M*/(*SD*)/Range	80.7/(7.8)/56–97
CR Female, n (%)	220 (55.4)
CR Educational Attainment, n (%)	
No schooling	133 (34.6%)
Primary	142 (37.0%)
Junior secondary	52 (13.5%)
Senior secondary or above	57 (14.9%)
CR MMSE score, *M*/(*SD*)	15.5/(6.2)
CG’s rating on CR’s health, n (%)	
Poor/fair	274 (69.4)
Good/very good/excellent	121 (30.6)
Affordability of living expenses, n (%)	
Not difficult at all	110 (28.1)
Not very difficult	149 (38.0)
Somewhat difficult	112 (28.6)
Very difficult	21 (5.4)

Notes. CG = caregiver, CR = care recipient, MMSE = Mini Mental State Examination.

**Table 2:** Descriptive statistics of Exemplary Care Scale (ECS) items and component loadings.

Items	Mean	SD	Skewness	% Missing ^a^	Component Loadings	
					Considerate Caregiving	Preserving Esteem
1. I make sure the food care recipient (CR) likes is available for meals and snacks. (FOOD)	2.09	0.90	−0.64	1.10	0.81	−0.18
2. To make (CR) feel refreshed and good about him/herself, I do things like being sure that he/she is dressed nicely or that his/her hair is clean and styled. (GROOM)	2.19	0.90	−0.84	1.97	0.81	−0.14
3. I make sure that where (CR) lives is bright and cheery. (BRIGHT ENVIRONMENT)	2.34	0.74	−1.05	0.88	0.80	−0.04
4. I try to maintain a relaxed, unhurried atmosphere for (CR). (RELAXED ATMOSPHERE)	2.14	0.82	−0.57	1.97	0.65	0.17
5. When at all possible, I make sure that (CR) gets to do some of the things that he/she enjoys (e.g., playing cards, visiting friends, going for a walk, listening to music). (HOBBY)	2.09	0.93	−0.71	2.63	0.53	0.22
6. I avoid being overcritical of (CR). (NOT OVERCRITICAL)	1.68	0.97	−0.01	0.44	−0.09	0.74
7. I really try to avoid interrupting (CR) when he/she is talking. (NOT INTERRUPT)	1.69	0.98	−0.03	1.75	0.07	0.66
8. I do everything I can to avoid making (CR) feel that he/she is a burden to me. (NOT A BURDEN)	1.78	1.02	−0.32	5.04	0.16	0.61
9. I actively avoid treating (CR) like a child. (NOT TREATING CHILD-LIKE)	1.26	1.04	0.41	2.19	−0.26	0.61
10. I take the time to sit and talk with (CR). (SIT AND TALK)	1.51	0.93	0.23	0.00	0.27	0.42
11. I make sure (CR) is included in special gatherings such as family and friends getting together or holidays when at all possible. (GATHERING)	1.73	1.03	−0.10	0.88	0.22	0.37

*Notes. N* = 397. Boldface indicates highest factor loadings. Responses for each items: 0 = *never*, 1 = *sometimes*, 2 = *often*, 3 = *always.* ^a^ Percentage of missing cases on the item out of the total sample of 456 participants.

**Table 3:** Correlations of ECS and its subscales with CR variables.

Variables	M (SD)	Possible Range	Correlations		
			ECS	Considerate Caregiving	Preserving Esteem
1. Memory PB Occurrence	2.39 (0.97)	0 to 3	0.01	0.06	−0.03
2. Disruption PB Occurrence	0.82 (1.11)	0 to 3	−0.06	−0.02	−0.08
3. Mood PB Occurrence	0.82 (1.06)	0 to 3	0.04	0.05	0.03
4. MMSE	15.54 (6.19)	0 to 30	0.13 *	0.11 *	0.11 *
5. Caregiver-rated health	1.12 (0.83)	0 to 4	0.07	−0.00	0.13 *

*Notes.* PB = Problem behavior; MMSE = Mini Mental State Examination. * *p* < 0.05.

**Table 4:** Correlations of ECS and its subscales with caregiver variables.

Variables	M (SD)	Possible Range	Correlations		
			ECS	Considerate Caregiving	Preserving Esteem
1. Depressive symptoms	15.30 (9.20)	0 to 60	−0.20 **	−0.15 **	−0.21 **
2. Caregiving burden	19.71 (8.84)	0 to 48	−0.13 *	−0.03	−0.20 **
3. Positive aspects of caregiving	26.97 (9.94)	0 to 44	0.25 **	0.24 **	0.22 **
4. Memory PB Bother	1.83 (1.22)	0 to 4	−0.18 **	−0.11 *	−0.20 **
5. Disruption PB Bother	2.05 (1.22)	0 to 4	−0.16 *	−0.11	−0.17 *
6. Mood PB Bother	1.87 (1.15)	0 to 4	−0.06	−0.06	−0.05
7. Memory PB Confidence	1.33 (1.08)	0 to 4	0.15 **	0.15 **	0.11 *
8. Disruption PB Confidence	1.22 (1.01)	0 to 4	0.13	0.12	0.12
9. Mood PB Confidence	1.19 (1.03)	0 to 4	0.21 **	0.20 **	0.18 *
10. IADL load	5.68 (2.22)	0 to 8	−0.08	−0.03	−0.10 *
11. ADL load	1.72 (2.00)	0 to 6	0.01	0.08	−0.06
12. IADL frustration	4.33 (5.38)	0 to 32	−0.10	−0.04	−0.13 *
13. ADL frustration	1.98 (3.48)	0 to 24	−0.07	−0.01	−0.11 *
14. Social support	4.70 (1.86)	0 to 8	0.14 **	0.14 **	0.11 *
15. Felt like screaming or yelling ^a^	0.78 (0.79)	0 to 3	−0.12 *	−0.07	−0.14 **
16. Had to keep yourself from hitting or slapping ^a^	0.15 (0.44)	0 to 3	−0.13 *	−0.07	−0.15 **

*Notes.* PB = Problem behavior; IADL = instrumental activities of daily living; ADL = activities of daily living. ^a^ Both items are potentially harmful behaviors. * *p* < 0.05, ** *p* < 0.01.

## Abbreviations

ADL	Activity of daily living
BPSD	Behavioral and psychological symptoms of dementia
C-ECS	Proto-oncogene tyrosine-protein kinase
NSCLC	Chinese version of Exemplary Care Scale
CESD	Center for Epidemiologic Studies Depression Scale
EC	Exemplary care
ECS	Exemplary Care Scale
MMSE	Mini Mental State Examination
NGO	Non-governmental organization
PAC	Positive aspects of caregiving
PHB	Potentially harmful behaviors
PWD	Person with dementia
ZBI	Zarit Burden Interview
